# METS-IR as an important predictor of neurological impairment severity in patients with severe cerebral infarction: a multicenter study based on the Chinese population

**DOI:** 10.3389/fneur.2024.1450825

**Published:** 2024-09-25

**Authors:** Yaqi Hou, Xiaohua Wu, Yiheng Shi, Xiaotian Xu, Yu Zhang, Lei Jiang, Wei Wang, Yan Yang, Lanying Hu

**Affiliations:** ^1^School of Nursing, Yangzhou University, Yangzhou, Jiangsu, China; ^2^Department of Endocrinology and Hematology, Affiliated Hospital of Yangzhou University, Yangzhou, Jiangsu, China; ^3^Department of Gastroenterology, The Affiliated Hospital of Xuzhou Medical University, Xuzhou Jiangsu, China; ^4^Department of Neurology, Affiliated Hospital of Yangzhou University, Yangzhou, Jiangsu, China; ^5^Department of Nursing, Affiliated Hospital of Yangzhou University, Yangzhou, Jiangsu, China

**Keywords:** METS-IR, cerebral infarction, neurological function, ICU, NIHSS

## Abstract

**Background:**

Insulin resistance (IR) is linked to an increased risk of neurological impairment following a stroke and may contribute to poor neurological prognosis in affected patients. The metabolic score for the insulin resistance index, shortened as the METS-IR, generally serves as a surrogate index for IR. However, its association with the severity of neurological impairment in patients with severe cerebral infarction (CI) in neurological intensive care units (ICU) has not been fully established.

**Methods:**

Patients with a diagnosis of CI, admitted to the neurological ICUs of Yangzhou University’s Affiliated Hospital and Xuzhou Medical University’s Affiliated Hospital, were included in the study. A multivariate logistic regression model and restricted cubic splines (RCS) were employed to explore the relationship between the METS-IR index and the severity of neurological impairment in these patients. The predictive capabilities of the METS-IR index and the triglyceride-glucose (TyG) index for outcome measures were compared through the ROC curve. Furthermore, a decision curve analysis was executed, and the integrated discrimination improvement (IDI) index was computed to evaluate the enhancements in predictive performance and clinical utility of various scoring systems with the inclusion of the METS-IR index. Subgroup analysis was conducted regarding age, BMI, and smoking status.

**Results:**

The study ultimately included 504 participants. Adjusted logistic regression and RCS results showed that as the METS-IR index increases, the risk of neurological impairment in patients with severe CI consistently grows (*P* for overall = 0.0146, *P*-nonlinear: 0.0689). The METS-IR index’s predictive capability for neurological impairment (AUC = 0.669) was superior to that of the TyG index (AUC = 0.519).

**Conclusion:**

From the study results, the METS-IR index can serve as an important predictor for neurological impairment in ICU patients with severe CI. It can aid in the identification and early intervention of neurological impairment in these patients.

## 1 Introduction

Stroke is a significant contributor to the global disease burden (1). According to the 2022 report by the World Stroke Organization (WSO), globally, stroke is the second leading cause of mortality and the third leading cause of disability (2), with ischemic stroke accounting for 75–80% of cases (3). Acute cerebral infarction (ACI), a subtype of ischemic stroke, results from necrosis or softening of brain tissue due to ischemia and hypoxia. Most survivors of cerebrovascular events suffer from disabilities and varying degrees of neurological dysfunction, including hemiplegia, aphasia, dysphagia, and cognitive disturbance (4). Patients with cerebrovascular diseases admitted to the ICU frequently exhibit more severe disturbances of consciousness, more complex conditions, and higher mortality (5). Clinical treatment regimens primarily include optimization of cerebral circulation, neuroprotection, intracranial pressure regulation, and intravenous thrombolysis (6, 7). Despite these therapeutic methods, the irreversibility of neuronal loss can lead to increased disability and recurrence rates associated with cerebral infarction (CI), posing a threat to patient survival (8). Therefore, the use of effective neurological function assessment tools is of paramount importance for the optimization of the management and implementation of preventive strategies for patients with severe CI.

Insulin resistance (IR) can be defined as the diminished effectiveness of insulin in facilitating the uptake and utilization of glucose, which is a prominent feature of metabolic syndrome (9). It affects metabolic signaling pathways in multiple organs (10), contributing significantly to the progression of vascular dysfunction. Additionally, IR is a significant factor in the progression of arterial stiffness, metabolic syndrome, as well as endothelial dysfunction, and it also serves as a risk factor for the advancement of stroke. Prior research has demonstrated that IR is independently related to adverse clinical outcomes of ischemic stroke (11, 12). It can exacerbate neurological deterioration during hospitalization and trigger ischemic stroke recurrence. The Euglycemic-Hyperinsulinemic Clamp (EHC), in the 1970s, was established as the gold standard for assessing IR (13). However, its clinical application is limited due to its invasiveness and cost. In a recent study, Bello-Chavolla et al. (14) introduced a novel assessment tool: the metabolic score for insulin resistance (METS-IR) index. This index, designed to evaluate insulin sensitivity, integrates measurements of fasting blood glucose (FBG), fasting triglycerides (TG), high-density lipoprotein cholesterol (HDL-C), as well as body mass index (BMI). This index is now considered a more accurate tool for evaluating insulin sensitivity. As a surrogate indicator for IR, METS-IR is associated with a variety of cardiovascular incidents, such as diabetes mellitus and ischemic heart disease. Also, its predictive function in assessing inflammatory activities and endothelial dysfunction has been underscored (14, 15). In the field of cerebrovascular research, the METS-IR index is widely used to investigate the correlation between stroke subtypes and outcomes, post-stroke mortality, and other related factors (16). However, the discussion on METS-IR and neurological impairment in patients with CI is scarce.

Therefore, this study was carried out to explore whether the METS-IR index could serve as a predictor for neurological impairment in ICU patients with severe CI. Identifying this association may facilitate the early identification of high-risk patients, allowing for more focused monitoring or preemptive interventions.

## 2 Methods

### 2.1 Data collection

Data were retrospectively gathered from patients diagnosed with CI who were admitted to the Neurological ICU at both the Affiliated Hospital of Yangzhou University and the Affiliated Hospital of Xuzhou Medical University between January 2020 and December 2023. Inclusion criteria: (1) Met the WHO diagnostic criteria for CI, confirmed by CT or MRI scans; and (2) Aged 18 years and above. Exclusion criteria: (1) Not admitted to the ICU; (2) Absence of National Institutes of Health Stroke Scale (NIHSS) score data; (3) Missing baseline data for FBG, HDL-C, TG, or BMI; (4) Presence of malignant tumors, cerebral hemorrhage, or other severe diseases; and (5) Missing data on other confounders. Ultimately, 505 eligible patients with severe CI were included. This study adhered to the Declaration of Helsinki and received ethical approval from the Ethics Committee of Yangzhou University (Ethical code: YZUHL20230091). Prior to the commencement of the study, all subjects or their legally authorized representatives provided their informed consent.

### 2.2 Data extraction

Baseline data collection was performed by professional researchers, who extracted basic patient information, drug characteristics, medical history, laboratory indicators, and other information, from the patient’s medical records. Collected basic demographics included age, sex, weight, height, history of smoking, as well as the consumption of alcohol; medication use included antiplatelet drugs, hypolipidemic drugs, and anticoagulants; medical history covered the history of diabetes mellitus, hypertension, and coronary heart disease. Laboratory indicators measured included fibrinogen (FIB), platelet count (PLT), total cholesterol (TC), TG, HDL-C, low-density lipoprotein cholesterol (LDL-C), creatinine (Cr), urea (UN), uric acid (UA), FBG, lactate dehydrogenase (LDH), creatine kinase (CK), and creatine kinase-myoglobin binding (CKMB). Assessment tools utilized were the NIHSS and the Glasgow Coma Scale (GCS). The calculation of the METS-IR index employed the following formula: Ln [(2 × FBG (mg/dL)) + TG (mg/dL)] × BMI (kg/m^2^)/Ln[HDL-C (mg/dL)]. For laboratory indicators assessed repeatedly within the first 24 h of admission, the first recorded value after admission was used. In order to mitigate bias stemming from the exclusion of samples, the proportion of missing values for each continuous variable was determined. For variables with less than a 20% share of missing values, multiple imputation was employed to predict missing values. Variables exhibiting more than a 20% rate of missing values were excluded from the analysis.

### 2.3 Definition of outcome measures

The NIHSS was used to assess neurologic function in patients with severe CI (17). The severity of stroke can be classified as normal or near-normal (0–1 points), mild (1–4 points), moderate (5–15 points), moderate-to-severe (15–20 points), and severe (21–42 points). A higher NIHSS score is indicative of greater neurological impairment. Since the patients in our sample were drawn from the neuro ICU, where stroke severity is generally high, in this study, an NIHSS score of 15 or higher was defined as severe neurological impairment. Accordingly, patients were divided into two groups: those with less severe neurological impairment (NIHSS <15) and those with severe neurological impairment (NIHSS ≥15).

### 2.4 Statistical analysis methods

The Shapiro–Wilk test was applied to assess the normality of continuous variables. Continuous variables that adhered to a normal distribution were expressed as mean ± standard deviation (SD), while those that did not were expressed as median (interquartile range [IQR]). Categorical variables were expressed as frequencies and percentages (%). For inter-group comparisons, Pearson’s chi-square test or t-tests were utilized. To identify and select relevant feature variables, the Least Absolute Shrinkage and Selection Operator (LASSO) method was applied. This method minimizes prediction error while selecting the most relevant variables to reduce model overfitting and identify variables that contribute to the outcomes. For cross-validation results, lambda = 1se was adopted to determine the covariates to be included. The logistic regression model was employed to estimate odds ratios (ORs) and their 95% CIs. Adjustments for covariates based on different models (three models) were also made to verify the robustness of the results. We categorized the METS-IR index as a categorical variable based on tertiles, using the lowest tertile (Q1) as the reference group. Additionally, a restricted cubic spline (RCS) analysis was performed to investigate potential linear relationships between METS-IR index levels and outcomes. The area under the ROC curve (AUC_ROC) was calculated to assess the predictive accuracy of the METS-IR index and TYG index. DeLong’s test was used to examine the difference in AUC between METS-IR and TYG. The integrated discrimination improvement (IDI) was computed, and a decision curve analysis was performed. This analysis focused on the enhancement of the predictive capability and clinical value of GCS with the inclusion of the METS-IR index.

All participant data were subjected to subgroup analysis. To minimize the impact of outliers, all covariates (except those used for stratification) were adjusted in the model. Subgroup analysis was conducted based on smoking status (yes, no), age (<65, 65–74, ≥75), and BMI (<24, ≥24). The likelihood ratio test (LRT) was used to investigate interactions between METS-IR and other variables. Statistical significance was defined when the *p* value was less than 0.05 (two-sided), and all statistical analyses were conducted with R v4.2.2.

## 3 Results

### 3.1 Clinical characteristics of patients

Among the 504 patients analyzed, the median age was 73.00 [64.00, 80.00] years, with 54.6% males and 45.4% females. Based on the NIHSS score, participants were divided into two groups: those with severe neurological impairment (272 patients) and those with less severe neurological impairment (232 patients). CI patients with severe neurological impairment had higher ages, BMI, systolic blood pressure, TG, HDL-C, UN, Cr, FBG, LDH, and CK; in addition, the incidence of heart disease was also obviously higher in these patients than that in the mild neurological impairment group (*p* < 0.05) (Table 1).

**Table 1:** Clinical baseline characteristics of patients.

	Level	Overall	NIHSS < 15	NIHSS ≥ 15	*p*
*n*		504	232	272	
Age		73.00 [64.00, 80.00]	71.00 [59.00, 78.00]	75.00 [68.00, 80.00]	<0.001
Sex (%)	Male	275 (54.6)	135 (58.2)	140 (51.5)	0.156
	Female	229 (45.4)	97 (41.8)	132 (48.5)	
BMI		25.10 [23.51, 27.06]	24.22 [22.22, 25.97]	25.95 [24.22, 27.55]	<0.001
Systolic pressure (mmHg)		153.00 [139.75, 172.00]	151.00 [137.75, 167.00]	154.00 [142.00, 179.00]	0.021
Diastolic pressure (mmHg)		88.00 [78.00, 99.00]	87.00 [78.00, 98.25]	89.00 [79.75, 100.00]	0.26
Smoking history (%)	No	380 (75.4)	178 (76.7)	202 (74.3)	0.593
	Yes	124 (24.6)	54 (23.3)	70 (25.7)	
Drinking history (%)	No	403 (80.0)	188 (81.0)	215 (79.0)	0.657
	Yes	101 (20.0)	44 (19.0)	57 (21.0)	
History of diabetes mellitus (%)	No	364 (72.2)	161 (69.4)	203 (74.6)	0.227
	Yes	140 (27.8)	71 (30.6)	69 (25.4)	
History of hypertension (%)	No	127 (25.2)	56 (24.1)	71 (26.1)	0.687
	Yes	377 (74.8)	176 (75.9)	201 (73.9)	
History of coronary heart disease (%)	No	426 (84.5)	206 (88.8)	220 (80.9)	0.02
	Yes	78 (15.5)	26 (11.2)	52 (19.1)	
FIB (g/L)		3.10 [2.53, 3.97]	2.99 [2.53, 3.71]	3.25 [2.49, 4.22]	0.085
PLT (*10^9/L)		188.50 [149.00, 235.00]	185.50 [152.75, 235.00]	190.00 [149.00, 235.00]	0.809
TC (mmol/L)		4.24 [3.41, 5.16]	4.27 [3.42, 5.18]	4.18 [3.40, 5.06]	0.529
TG (mmol/L)		1.06 [0.80, 1.45]	1.17 [0.87, 1.60]	1.00 [0.73, 1.32]	<0.001
HDL-C (mmol/L)		1.17 [0.97, 1.43]	1.15 [0.96, 1.36]	1.19 [1.00, 1.45]	0.037
LDL-C (mmol/L)		2.39 [1.84, 3.19]	2.40 [1.82, 3.21]	2.39 [1.85, 3.16]	0.83
UN (mmol/L)		6.04 [4.64, 7.73]	5.70 [4.38, 7.25]	6.36 [4.94, 8.69]	0.001
Cr (umol/L)		65.05 [53.98, 78.40]	63.00 [51.68, 76.00]	67.00 [55.98, 82.67]	0.014
UA (umol/L)		281.30 [223.00, 377.70]	292.85 [233.00, 392.48]	274.00 [216.20, 363.97]	0.023
FBG (mmol/L)		6.20 [5.50, 7.88]	5.80 [5.20, 6.32]	7.00 [5.82, 9.00]	<0.001
LDH (U/L)		226.00 [194.75, 275.25]	217.00 [185.00, 252.05]	238.00 [201.00, 287.00]	<0.001
CK (U/L)		93.00 [64.00, 167.50]	85.50 [57.75, 130.50]	101.50 [70.00, 200.55]	0.001
CKMB (U/L)		6.86 [2.86, 13.00]	7.00 [3.00, 13.00]	5.63 [2.82, 12.00]	0.335
Antiplatelet drug (%)	No	62 (12.3)	8 (3.4)	54 (19.9)	<0.001
	Yes	442 (87.7)	224 (96.6)	218 (80.1)	
Hypolipidemic drug (%)	No	39 (7.7)	8 (3.4)	31 (11.4)	0.002
	Yes	465 (92.3)	224 (96.6)	241 (88.6)	
Anticoagulant (%)	No	398 (79.0)	203 (87.5)	195 (71.7)	<0.001
	Yes	106 (21.0)	29 (12.5)	77 (28.3)	
GCS		11.00 [8.00, 14.00]	14.00 [12.00, 15.00]	8.00 [7.75, 11.00]	<0.001

### 3.2 Feature variable screening

**Figure 1:**
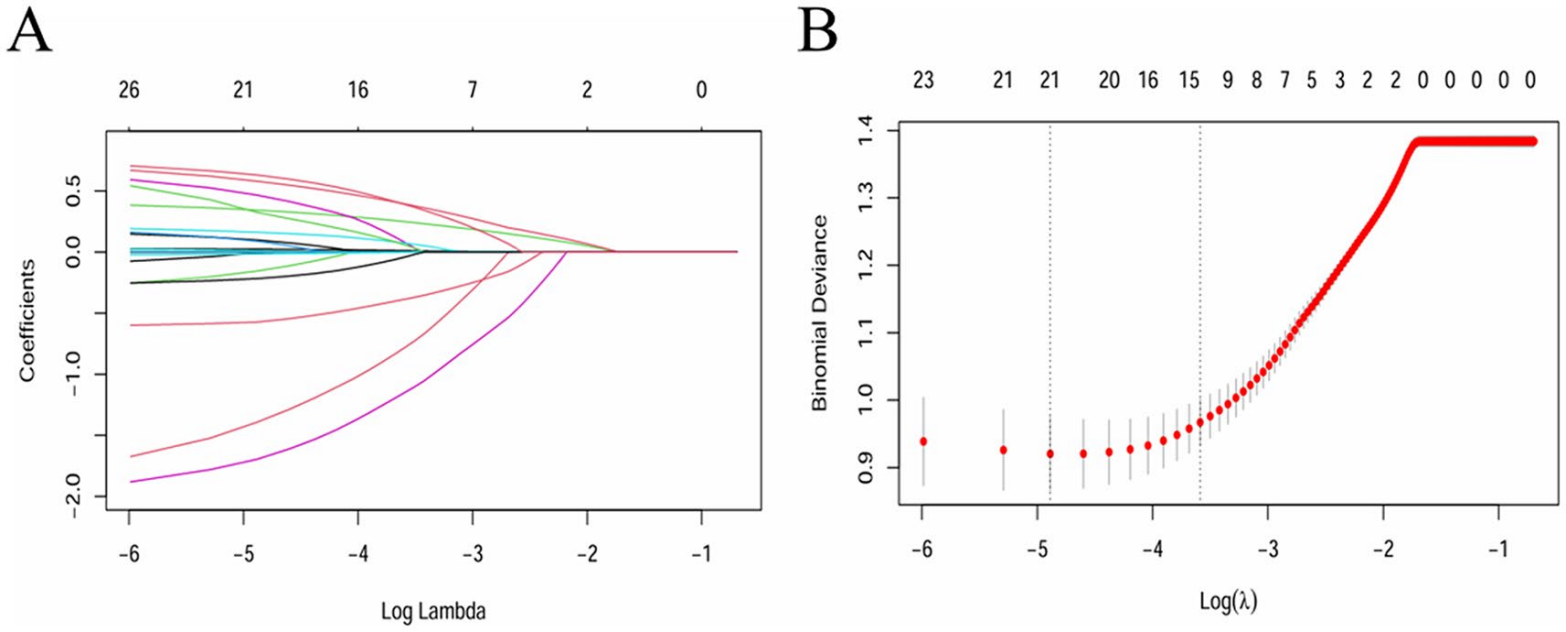
Feature variable screening using the LASSO regression (lambda = 1se was adopted to determine the covariates to be included). **(A)** Coefficient diagram of LASSO variables. **(B)** Optimal parameter (lambda) selection in the LASSO model.

LASSO regression was adopted to select covariates, which could minimize prediction error while simultaneously screening the most relevant variables, thereby reducing model overfitting and identifying variables that contribute to the outcomes. As per the LASSO regression (Figures 1A,B), lambda = 1se (lambda = 0.02763819) was selected to determine the included covariates, and 14 covariates, including age, BMI, FIB, TG, HDL-C, UA, FBG, LDH, CKMB, smoking history, history of diabetes mellitus, use of antiplatelet drugs, use of hypolipidemic drugs, and use of anticoagulants, were selected as the influencing factors of neurological impairment in ICU patients with severe CI (Supplementary Table S1). In addition, although sex was not significant in the LASSO analysis, considering the important impact of sex on neurological function impairment in patients (18, 19), sex was included as a covariate along with the factors identified through LASSO regression analysis in subsequent analyses.

### 3.3 Impact of METS-IR index on the degree of neurological impairment

The influence of exposure variables on outcome measurements was assessed, with covariates adjusted using a logistic regression model (Table 2): Model 1 was unadjusted; Model 2 was adjusted regarding age, sex and BMI; Model 3 was adjusted regarding age, sex, BMI, FIB, TG, HDL-C, UA, FBG, LDH, CKMB, smoking history, history of diabetes mellitus, use of antiplatelet drugs, use of hypolipidemic drugs, and use of anticoagulants.

**Table 2:** Logistic regression analysis of neurological impairment in patients with severe cerebral infarction.

	Model 1		Model 2		Model 3	
	OR (95%CI)	*p*	OR (95%CI)	*p*	OR (95%CI)	*p*
METS-IR	1.093 (1.065–1.124)	<0.001	1.078 (1.044–1.115)	<0.001	1.257 (1.163–1.368)	<0.001
Q1	–	–	–	–	–	–
Q2	2.064 (1.339–3.202)	0.001	1.444 (0.864–2.406)	0.200	2.134 (0.992–4.657)	0.054
Q3	3.431 (2.199–5.406)	<0.001	2.328 (1.344–4.047)	0.003	4.637 (1.685–13.19)	0.003

Model 1: Unadjusted.

Model 2: Adjusted for age, sex, and BMI.

Model 3: Further adjusted for smoking history, history of diabetes mellitus, FIB, TG, HDL-C, UA, FBG, LDH, CKMB, antiplatelet drug, hypolipidemic drug, anticoagulant based on Model 2.

The logistic regression analysis showed that in the unadjusted Model 1, patients with a higher METS-IR index had a positively correlated risk of experiencing neurological deficits (OR [95% CI], 1.093 [1.065, 1.024], *p* < 0.001). In Model 2, which was adjusted regarding demographics including age, sex and BMI, and in Model 3, which was fully adjusted regarding confounders, the risk of neurological deficits remained significantly higher in patients with a higher METS-IR index compared to those with a lower index (Model 2: OR [95% CI], 1.078 [1.044, 1.115], *p* < 0.001; Model 3: OR [95% CI], 1.257 [1.163, 1.386], *p* < 0.001).

When treated as a categorical variable, the results indicated that a higher METS-IR index was significantly associated with the severity of neurological function impairment in patients. In the unadjusted Model 1 (Q2: OR [95% CI], 2.064 [1.339, 3.202], *p* = 0.001; Q3: OR [95% CI], 3.431 [2.199, 5.406], *p* < 0.001), and in Model 2 adjusted for age, sex, and BMI (Q2: OR [95% CI], 1.444 [0.864, 2.406], *p* = 0.200; Q3: OR [95% CI], 2.328 [1.344, 4.047], *p* = 0.003), after full adjustment for confounders (Q2: OR [95% CI], 2.134 [0.992, 4.657], *p* = 0.054; Q3: OR [95% CI], 4.637 [1.685, 13.19], *p* = 0.003), patients in the Q3 METS-IR tertile had a significantly higher risk of in-hospital mortality compared to those in Q1.

**Figure 2:**
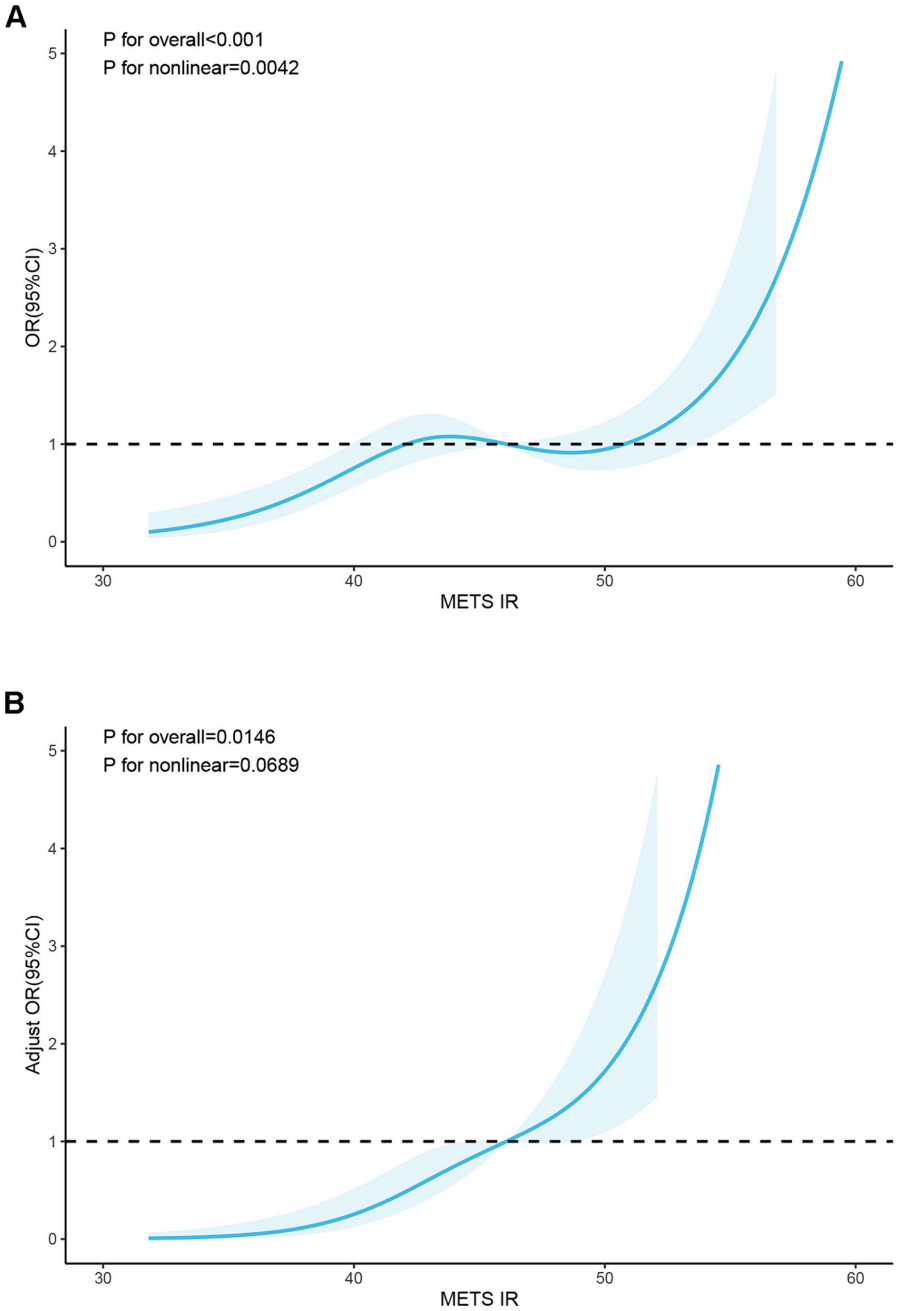
RCS curve of METS-IR index and neurological impairment in patients with severe CI. **(A)** Before adjusting for confounding factors, P for overall<0.001; P for nonlinear = 0.0042. **(B)** After adjusting for confounding factors, P for overall = 0.0146; P for nonlinear = 0.0689. (Confounders: Age, Sex, BMI, Smoking history, History of diabetes mellitus, FIB, TG, HDL_C, UA, FBG, LDH, CKMB, Antiplatelet drug, Hypolipidemic drug, Anticoagulant).

RCS analysis was performed to explore potential linear relationships between the METS-IR index and clinical outcomes. RCS results showed that before adjusting for confounders, the relationship between the METS-IR index and neurological impairment in patients with severe CI was generally significant, although it exhibited a nonlinear trend (Figure 2A). Specifically, when the METS-IR index surpassed a value of 50, there was a significant escalation in the risk of neurological impairment (*P* for overall <0.001, *P*-nonlinear = 0.0042). However, after adjusting for confounders, the relationship between the METS-IR index and neurological impairment shifted to a linear pattern (Figure 2B). The risks of neurological impairment increased progressively with the rise in the METS-IR index (*P* for overall = 0.0146, *P*-nonlinear = 0.0689).

### 3.4 Predictive capability and incremental effect of the METS-IR index

**Figure 3:**
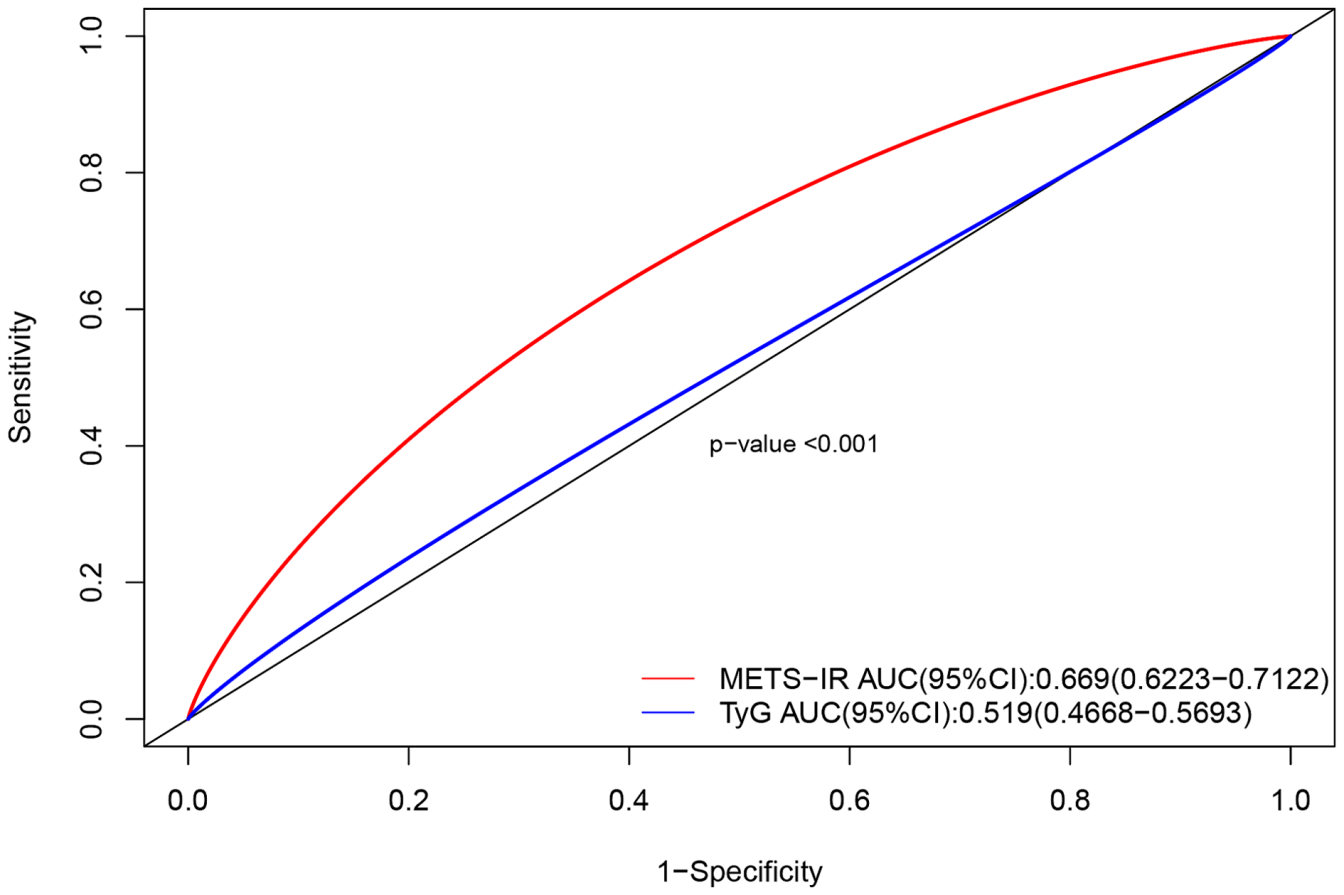
Comparison of ROC curves for the METS-IR index and TyG index in predicting neurological impairment in patients with severe CI.

The predictive performance of the METS-IR index was assessed by calculating the ROC_AUC for neurological impairment in ICU patients with severe CI. Meanwhile, the predictive performance of the TyG index and the METS-IR index was compared. The results revealed that the METS-IR index predicted neurological impairment with an AUC of 0.669 (95% CI: 0.6223–0.7122). Under the same model, we also calculated the ability of the TyG index to predict neurological impairment in patients with severe CI; it yielded an AUC of 0.519 (95% CI, 0.4668–0.5693). The difference between the two ROC curves was statistically significant (*p* < 0.001) (Figure 3). Thus, the METS-IR index demonstrated superior predictive capability for the risk of neurological impairment in patients with severe CI compared to the TyG index.

**Figure 4:**
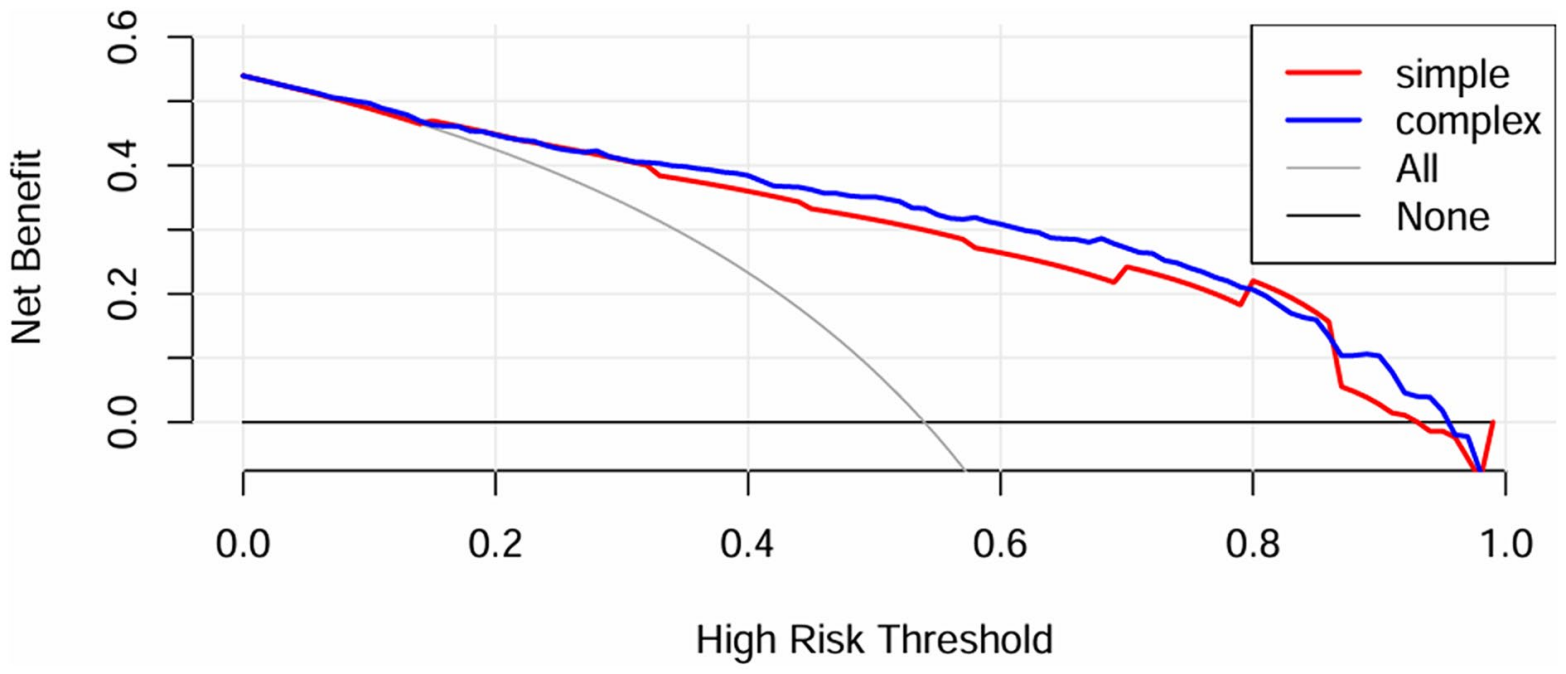
DCA for predicting neurological impairment in patients with severe CI.

Furthermore, the IDI for the GCS was calculated to analyze the impact on the predictive capability of the scoring tools with the inclusion of the METS-IR index. IDI is an assessment tool used to gauge the enhancement in model predictive capability, where values above zero indicate improvement and values below zero indicate a negative result. The results indicated that the predictive capability of the scoring tools improved with the inclusion of the METS-IR index (IDI [95% CI]: 0.0516 [0.0329–0.0704], *p* < 0.05). Additionally, a DCA (Figure 4) was performed to assess the improvement in clinical utility with the inclusion of the METS-IR index. The results demonstrated an improvement in the net clinical benefit of the scoring tools with better clinical applicability, after the METS-IR index was added.

### 3.5 Subgroup analysis

Subgroup analysis was performed separately based on patient age, sex, BMI, and smoking history (Table 3). In the subgroup analysis, all covariates (except for the stratifying covariate) were adjusted for in the model. The results showed that no interaction was found between age, BMI, or sex and the METS-IR index (*P*-interaction >0.05). However, a significant interaction was observed between smoking history and the METS-IR index (*P*-interaction = 0.035). A higher METS-IR index was associated with an increased risk of neurological impairment in smokers (OR [95%CI], 1.263[1.065, 1.541]), compared to non-smokers (OR [95% CI], 1.278[1.166, 1.413]). The METS-IR index remained a significant predictor among non-smokers and across all BMI classifications (<24 OR [95% CI]: 1.286 [1.075, 1.587]; ≥24 OR [95% CI]: 1.261 [1.149, 1.396]) and in both males (OR [95% CI]: 1.315 [1.171, 1.498]) and females (OR [95% CI]: 1.277 [1.142, 1.449]) (*p* < 0.05).

**Table 3:** Association between METS-IR and neurological impairment in patients with severe cerebral infarction in different subgroups.

Subgroup	OR (95%CI)	*p*	P-interaction
Smoking history			0.035
No	1.278 (1.166, 1.413)	<0.001	
Yes	1.263 (1.065, 1.541)	0.012	
Age			0.400
<65	1.269 (1.080, 1.538)	0.008	
65–74	1.204 (1.038, 1.419)	0.019	
≥75	1.243 (1.101, 1.427)	<0.001	
BMI			0.200
<24	1.286 (1.075, 1.587)	0.010	
≥24	1.261 (1.149, 1.396)	<0.001	
Sex			0.089
Male	1.315 (1.171, 1.498)	<0.001	
Female	1.277 (1.142, 1.449)	<0.001	

All covariates (except those used for stratification) were adjusted for in the model.

## 4 Discussion

This study marks the first to demonstrate that the METS-IR index can effectively predict neurological impairment in ICU patients with severe CI. After accounting for potential confounders, a clear positive linear correlation emerged between the METS-IR index and neurological impairment in this patient group. Moreover, the METS-IR index outperformed the TyG index in predicting neurological impairment in these patients. Additionally, when compared to certain traditional risk factors, the inclusion of METS-IR enhanced the predictive and discriminative capabilities for neurological impairment in patients with severe CI.

METS-IR has recently been recognized as a novel, reliable, straightforward indicator for IR. Research has demonstrated its utility in screening for early insulin sensitivity and diseases related to metabolism (20). A prospective cohort study exploring the relationship between the METS-IR index and the risk of new-onset T2DM in a non-obese Japanese population found that an elevated METS-IR index is independently linked to an increased risk of T2DM among these individuals (20). Additionally, a cohort study conducted in Korea revealed that elevated METS-IR can predict the future risk of ischemic heart disease in a non-diabetic community population, further establishing METS-IR as a valuable predictive biomarker for ischemic heart disease (21). A cross-sectional study in Japan showed that, the METS-IR index, when adjusted for potential confounders and used as both a continuous and categorical variable, was linearly correlated with arterial stiffness as measured by brachial-ankle pulse wave velocity (baPWV) in the Japanese health examination population (22). Arterial calcification is a significant marker of cardiovascular disease. It impacts not only the heart but also cerebrovascular health. A study investigating the relationship between the METS-IR index and coronary artery calcification (CAC) indicated that an increase in METS-IR is independently associated with a higher prevalence of CAC (23). Additionally, for individuals with diabetes mellitus, the cardiovascular risk is elevated, and METS-IR may play a critical role in mortality. A study based on the NHANES database demonstrated a non-linear relationship between METS-IR and all-cause and CVD-related mortality in patients with diabetes mellitus, where METS-IR below a certain threshold was inversely associated with all-cause and CVD-related mortality (24). Regarding the cerebrovascular research field, a large-scale, retrospective cohort study assessed the risk of stroke and its subtypes in relation to METS-IR. This study observed a near J-shaped association between the METS-IR level and the risk of both overall stroke and ischemic stroke specifically (16). Additionally, a prospective study involving 1,074 patients found that after adjusting for potential confounders, an elevated METS-IR index was linked to an increased risk of adverse outcomes among ischemic stroke patients after intravenous thrombolysis. In this analysis, the AUC of METS-IR in predicting poor prognosis was 0.790 (95% CI: 0.761–0.819) (25). In summary, the METS-IR index has shown favorable predictive performance for diabetes mellitus, cardiovascular diseases, and other conditions. There is potential for developing interventions based on the METS-IR index, which may offer several advantages.

Prior studies have recognized the TyG, TyG-BMI, TG/HDL-C, and METS-IR indices as simple and reliable markers for IR (14, 26–29). One particular study evaluated the efficacy of these four non-insulin-dependent IR indices in predicting the severity of coronary artery disease (CAD). The results indicated that, the TyG, TyG-BMI, TG/HDL-C, and METS-IR indices may be important predictors of CAD severity. Notably, among these indices, METS-IR was found to have the highest predictive value, with TyG-BMI following closely behind (30). Additionally, Bello-Chavolla et al. conducted an analysis highlighting the superior performance of METS-IR over biomarkers such as TyG and EHC in diagnosing impaired insulin sensitivity. Their findings indicated that the METS-IR is notably superior to other biomarkers (14). In this study, according to the collected clinical data, the AUC_ROC indicated that the METS-IR index outperformed the TyG index in the prediction of neurological impairment in ICU patients with severe CI (*p* < 0.05). The METS-IR comprises FBG, TG, HDL-C, and BMI. These indices are easily collected in clinical laboratories and can effectively assess insulin sensitivity. The METS-IR index is a feasible tool for predicting neurological impairment in clinical settings. Our study results suggested that an elevated METS-IR may assist in identifying neurological impairment in ICU patients with severe CI. From a clinical application perspective, contemporary e-medical record systems could potentially automate the calculation of METS-IR to enhance risk stratification. This capability allows for more targeted monitoring and early intervention for these patients. Future research could investigate and compare the effectiveness of different insulin resistance indices in predicting neurological impairment.

Through interaction investigation of this study, compared to the non-smoker, in smokers, there was a higher METS-IR index correlated with more severe neurological impairment in patients with severe CI (*P*-interaction = 0.033). Also, extensive studies have confirmed the detrimental effect of smoking on neurological function. A study (31) found that, over a long period, compared to the gas phase, free radicals in cigarette smoke tar accumulate from hours to months. These radicals are involved in the elevated Ca^2+^ and Na^+^ levels in cytoplasm and mitochondria. Excessive influx of Ca^2+^ into the mitochondria (excitotoxicity) can lead to neuronal impairment. Then, mitochondrial calcium overload can result in membrane expansion and rupture. In addition, the release of small molecules and macromolecules in the cytoplasm activates neurodegeneration. On the other hand, smoking significantly contributes to cardiovascular and cerebrovascular impairment. Smoking has been identified as a significant risk factor for stroke, particularly ischemic stroke. A comprehensive 14-year follow-up cohort study conducted in Japan showed that, compared to non-smokers, higher daily cigarette consumption was correlated with an increased risk of CI. However, the association with cerebral hemorrhage did not reach statistical significance (32).

IR is a systemic disease that affects multiple organs and insulin regulatory pathways, marked by increased insulin levels but diminished physiological effects. While this study elucidated the relationships between METS-IR and neurological impairment in patients with severe CI, the underlying mechanisms remain obscure. Existing literature suggested several potential mechanisms that could elucidate this association. One primary mechanism involves IR exacerbating pathophysiological processes within endothelial cells, smooth muscle cells, and macrophages. This exacerbation is mediated by inflammation, which subsequently fosters the formation of arteriosclerotic-associated foam cells and vulnerable plaques, thereby contributing to vascular complications that may precipitate neurological impairment (33–35). This phenomenon is closely related to cerebrovascular narrowing or occlusion and is associated with CI events. Following a stroke, there is an elevation in several pro-inflammatory cytokines associated with insulin resistance within the brain. In patients with elevated METS-IR, certain cytokines may provoke local inflammatory responses that lead to dysfunction of the blood–brain barrier. This disruption can further exacerbate ischemic neuronal impairment. Moreover, metabolic disturbances can result in increased blood viscosity and elevated cerebral water content, which subsequently reduces cerebral blood flow and leads to intracellular acidosis. In hyperglycemic patients, the accumulation of nitrogenous waste in the brain can inflict toxic and metabolic damage on brain tissues, notably affecting the basal ganglia. This damage impacts focal cellular metabolism and results in cellular edema, while also enhancing the permeability of the blood–brain barrier (36, 37). The severity of symptoms is closely related to the neurological impairment caused by the blood–brain barrier dysfunction.

This study has several advantages over previous research. Firstly, to the best of our knowledge, this is the first cross-sectional study assessing the association between METS-IR and neurological impairment in patients with severe CI. Secondly, this study reported results from real-world clinical practice. As a multicenter retrospective study that included real-world data from two major grade A tertiary hospitals, selection bias typical of single-center studies was mitigated to some extent. However, certain limitations are inevitable in this study. Firstly, the observational and retrospective study design restricted the inference of causal relationships. In terms of statistics, to control for confounders, a series of sensitivity analyses were conducted by LASSO regression. This method offers advantages in selecting highly explanatory variables, handling high-dimensional data, and addressing multicollinearity issues. It can compress the regression coefficients of some unnecessary variables to zero, thereby excluding them from the model, achieving the goal of variable selection. However, limitations exist, as LASSO regression may exclude some biologically significant variables with small effects, such as blood pressure, which can affect the model’s interpretability. Additionally, due to practical limitations, some potential confounders were not included in the study, such as the time from onset to hospital arrival, neuroimaging data, and mRs scores. Although some treatment information was included (such as the use of antiplatelet drugs, hypolipidemic drugs, and anticoagulants), other specific treatment methods were not captured. This may affect the results. In future research, comprehensive information collection should be strengthened to make the research results more generalizable and convincing. In addition, the participants of this study were primarily ICU patients with severe CI in China, resulting in a relatively small sample size. Whether our study’s findings can be generalized to severe CI patients who were not admitted to the ICU is an important area for future research. Subsequent efforts should be directed toward expanding the sample size to further validate the results. Lastly, while efforts were made to control for known confounders, the possibility of interference from unmeasured factors such as genetic susceptibility and environmental exposure, or inaccuracies in measuring other confounders, cannot be fully dismissed. Due to certain operational constraints, the data collected in this study may be limited. Understanding how changes in METS-IR over time influence clinical outcomes is a crucial area for further investigation. However, in our current study, we only collected baseline data from the initial measurement, which prevents us from addressing the changes in METS-IR over time. This represents a limitation of our research. In future studies, we plan to design a prospective study that will include data from multiple time points, allowing for a more comprehensive assessment of the dynamic relationship between METS-IR fluctuations and clinical outcomes.

## 5 Conclusion

Our study indicated that the METS-IR index is a key predictor of neurological impairment in patients with severe CI. This index exhibited a positive linear correlation with the risk of neurological impairment. Furthermore, METS-IR, a simple and reliable biomarker derived from non-insulin measurements, holds significant potential in predicting and preventing cerebrovascular diseases, which can enhance the accuracy of identifying high-risk patients. Future research could develop interventions based on the METS-IR index, which may have a notable effect on early management of neurological impairment in patients with severe CI.

## Data Availability

The original contributions presented in the study are included in the article/Supplementary material, further inquiries can be directed to the corresponding author.
