# Supplementary material for: METS-IR as an important predictor of neurological impairment severity in patients with severe cerebral infarction: a multicenter study based on the Chinese population
Source: Front Neurol. 2024 Sep 25;15:1450825. doi: 10.3389/fneur.2024.1450825 (PMC11461195; doi:10.3389/fneur.2024.1450825)
Supplement: Supplementary file 1 [file Table_1.docx]

| **Supplementary Table S1 Variables and coefficients identified by Lasso analysis when Lamda=1se** | |
| --- | --- |
| Variables | Coefficients |
| Age | 1.224686e-02 |
| Sex | . |
| BMI | 2.488755e-01 |
| Systolic pressure | . |
| Diastolic pressure | . |
| Smoking history | 7.771036e-02 |
| Drinking history | . |
| History of diabetes mellitus | -7.783473e-01 |
| History of hypertension | . |
| History of coronary heart disease | . |
| FIB | 7.336623e-02 |
| PLT | . |
| TC | . |
| TG | -3.849846e-01 |
| HDL-C | 5.782040e-02 |
| LDL-C | . |
| UN | . |
| Cr | . |
| UA | -5.071621e-05 |
| FBG | 3.935302e-01 |
| LDH | 6.302140e-04 |
| CK | . |
| CKMB | -1.951200e-03 |
| Antiplatelet drug | -1.146645e+00 |
| Hypolipidemic drug | -3.958237e-02 |
| Anticoagulant | 3.891416e-01 |
